# Prognostic Implications of High HFpEF Probability in Patients With Chronic Thromboembolic Pulmonary Hypertension

**DOI:** 10.1002/clc.70503

**Published:** 2026-11-05

**Authors:** Yijia Wang, Xin Li, Zhihua Huang, Anqi Duan, Qi Wang, Luyang Gao, Sicong Li, Qing Zhao, Tao Yang, Zhihui Zhao, Qin Luo, Zhihong Liu

**Affiliations:** ^1^ Center for Respiratory and Pulmonary Vascular Diseases, Fuwai Hospital, National Center for Cardiovascular Diseases, Chinese Academy of Medical Sciences and Peking Union Medical College Beijing China; ^2^ Department of Cardiology, Beijing Friendship Hospital Capital Medical University Beijing China

**Keywords:** chronic thromboembolic pulmonary hypertension, clinical worsening, heart failure with preserved ejection fraction, HFpEF‐ABA score

## Abstract

**Background:**

Chronic thromboembolic pulmonary hypertension (CTEPH) and left heart disease, particularly heart failure with preserved ejection fraction (HFpEF), show overlapping clinical features despite distinct pathophysiology.

**Objectives:**

The HFpEF‐ABA algorithm (age, body mass index, atrial fibrillation) was used to characterize HFpEF pretest probability in patients with CTEPH and evaluate its association with left heart disease and clinical worsening.

**Methods:**

Among 466 patients with CTEPH stratified by HFpEF‐ABA score (low < 25%, intermediate 25%–74%, high ≥ 75%), Spearman's correlation assessed associations with common left heart disease comorbidities. Kaplan‐Meier analysis and Cox regression evaluated associations with clinical worsening, with restricted cubic splines assessing the dose‐response relationship and non‐linearity. Subgroup analyses by sex, smoking, alcohol use, hypertension, coronary artery disease, and obstructive sleep apnea assessed the consistency. We also evaluated whether adding the HFpEF‐ABA score improved COMPERA 2.0 discrimination for clinical worsening.

**Results:**

Intermediate and high HFpEF probability was found in 52.4% (*n* = 244) and 9.0% (*n* = 42), respectively. After adjustment, each standard deviation increase in HFpEF probability was associated with an approximately 73% increase in clinical worsening (*p* < 0.001). Results were consistent for HFpEF probability (*p* < 0.05), with no significant subgroup interactions. Adding HFpEF‐ABA with COMPERA 2.0 improved discrimination, increasing the C‐index increasing from 0.532 to 0.634 (*p* = 0.012).

**Conclusions:**

Assessing HFpEF probability in patients with CTEPH identifies those at high risk for this comorbidity and improves the precision when integrated with existing risk models.

## Graphical abstract

Assessment of HFpEF probability using the HFpEF‐ABA algorithm identifies patients with chronic thromboembolic pulmonary hypertension at higher risk of clinical worsening. Adding HFpEF probability to COMPERA 2.0 improves prognostic discrimination and supports refined risk stratification.

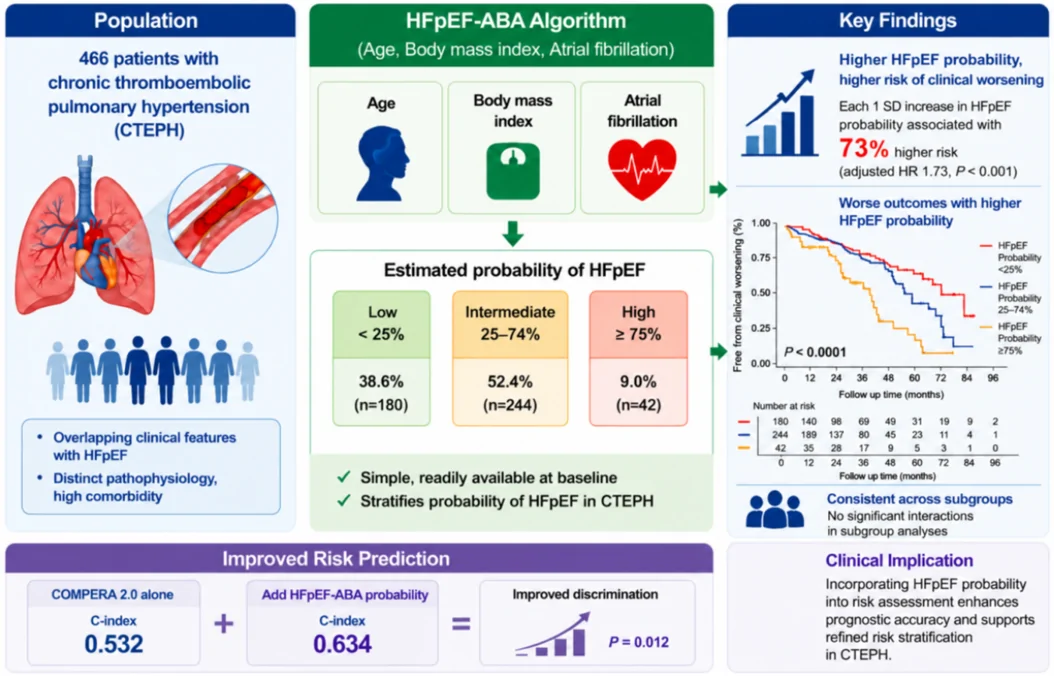

**Abbreviations**
AF: atrial fibrillation
BMI: body mass index
CAD: coronary artery disease
CI: confidence interval
CTEPH: chronic thromboembolic pulmonary hypertension
DM: diabetes mellitus
HFpEF: heart failure with preserved ejection fraction
HR: hazard ratio
IDI: integrated discrimination improvement
IQR: interquartile range
LHD: left heart disease
LVEF: left ventricular ejection fraction
mPAP: mean pulmonary arterial pressure
NRI: net reclassification improvement
NT‐proBNP: N‐terminal pro‐B‐type natriuretic peptide
OSA: obstructive sleep apnea
PAH: pulmonary arterial hypertension
PAWP: pulmonary artery wedge pressure
PH: pulmonary hypertension
PVR: pulmonary vascular resistance
SD: standard deviation

## 1 Introduction

Pulmonary hypertension (PH), defined by a mean pulmonary artery pressure > 20 mmHg at rest, comprises a heterogeneous group of disorders that may ultimately lead to right heart failure and death [1, 2]. Chronic thromboembolic pulmonary hypertension (CTEPH), classified as Group 4 PH, is caused by organized thromboembolic obstruction of the pulmonary arteries and secondary distal pulmonary microvasculopathy, resulting in increased pulmonary vascular resistance (PVR) and impaired cardiopulmonary function [3, 4]. In clinical practice, isolated CTEPH without relevant comorbidities is uncommon [5, 6], and many patients present with comorbidities related to left heart disease (LHD), including heart failure [7, 8, 9].

Heart failure with preserved ejection fraction (HFpEF) is a common clinical syndrome characterized by elevated left ventricular filling pressure and diastolic dysfunction. When HFpEF‐related features coexist with CTEPH, elevated left‐sided filling pressure may add a postcapillary component to the precapillary thromboembolic burden, leading to a more complex hemodynamic phenotype and potentially worse clinical outcomes. Therefore, identifying HFpEF probability in patients with CTEPH may provide additional information for risk stratification [8]. The H2FPEF score is widely used to estimate the probability of HFpEF, but its reliance on echocardiographic parameters may limit its applicability when complete imaging data are unavailable. The HFpEF‐ABA algorithm, based on age, body mass index (BMI), and atrial fibrillation (AF), provides a simple, noninvasive approach to estimate the pretest probability of HFpEF using routinely available clinical variables. This algorithm has demonstrated good performance in external validation cohorts [10]. Moreover, previous studies have shown that higher HFpEF probability assessed by HFpEF‐ABA is associated with worse outcomes in patients with pulmonary arterial hypertension (PAH) [11]. However, whether HFpEF probability has prognostic relevance in patients with CTEPH remains unclear.

Therefore, the aims of this study were to quantify the pretest probability of HFpEF in patients with CTEPH using the HFpEF‐ABA algorithm and to evaluate its association with clinical outcomes and incremental prognostic value beyond established CTEPH risk stratification, particularly COMPERA 2.0.

## 2 Methods

### 2.1 Study Design and Participants

This retrospective cohort study was conducted at Fuwai Hospital, and patients diagnosed with CTEPH between February 2014 and August 2023 were included. The inclusion criteria were as follows: (a) age ≥ 18 years; (b) complete baseline clinical and hemodynamic data, with a diagnosis of CTEPH based on current guidelines [1]; (c) available follow‐up data. Given that the HFpEF‐ABA score was applied in patients with left ventricular ejection fraction (LVEF) ≥ 50%, those with LVEF < 50% were excluded. For all individuals, HFpEF probability was calculated using the HFpEF‐ABA algorithm, and patients were divided into low (< 25%), intermediate (25%–74%), and high (≥ 75%) HFpEF probability groups [11]. This study was conducted according to the principles outlined in the Declaration of Helsinki and was ethically approved by the Ethics Committee of Fuwai Hospital (Approval No. 2024‐2441).

### 2.2 Data Collection and Definitions

Clinical data were abstracted from institutional electronic health records, including: (1) demographics and physical examination parameters; (2) laboratory tests [N‐terminal pro‐B‐type natriuretic peptide (NT‐proBNP), liver and kidney function, blood glucose and blood lipid indexes]; (3) echocardiography measures [left ventricular end‐diastolic diameter, LVEF, right ventricular end‐diastolic diameter, tricuspid annular plane systolic excursion, tricuspid regurgitation velocity]. Comorbidities were defined according to contemporary guidelines: hypertension as blood pressure ≥ 140/90 mmHg or antihypertensive treatment [12]; diabetes mellitus (DM) as HbA1c ≥ 6.5% or glucose‐lowering therapy [13]; coronary artery disease (CAD) was defined as ≥ 50% luminal narrowing in any vessel in the left main coronary artery, anterior descending coronary artery, circumflex coronary artery, right coronary artery and its important branches in previous coronary angiography [14]; obstructive sleep apnea (OSA) as having an obstructive predominant respiratory event ≥ 5 times/h during monitoring as confirmed by polysomnography or portable sleep monitoring [15]; BMI was calculated using WHO‐standardized methodology (kg/m^2^). Obesity was defined as BMI > 30 kg/m^2^ [16]. The HFpEF‐ABA score—a validated predictor integrating age, BMI, and AF status—was computed as previously described [11], with probability thresholds (< 25%, 25%–74%, ≥ 75%) corresponding to low, intermediate, and high pretest probability of HFpEF.

### 2.3 Hemodynamic Assessment

Right heart catheterization was performed under resting conditions using a Swan‐Ganz catheter via femoral or jugular venous access. Pressure measurements included right atrial pressure, pulmonary artery systolic/diastolic pressures, mean pulmonary arterial pressure (mPAP), and pulmonary artery wedge pressure (PAWP). Cardiac output was determined by thermodilution technique with triplicate measurements. Cardiac index was calculated as cardiac output and body surface area. PVR was calculated using the formula: PVR = (mPAP − PAWP)/cardiac output, expressed in wood units. All procedures adhered to 2022 European Society of Cardiology/European Respiratory Society technical standards for PH diagnosis [1].

### 2.4 Outcomes and Follow‑Up

The endpoint was clinical worsening, defined as the first occurrence of any of the following events: all‐cause mortality, unplanned rehospitalization due to worsening pulmonary hypertension or right heart failure, or escalation of targeted pulmonary hypertension therapy. Unplanned rehospitalization was defined as an unscheduled inpatient or emergency admission due to clinical worsening related to right heart failure or pulmonary hypertension, such as worsening dyspnea, edema, syncope, or signs of low cardiac output. Planned admissions for routine follow‐up, scheduled examinations, treatment evaluation, or elective procedures were not considered clinical worsening events. Escalation of targeted therapy was clarified as initiation of parenteral prostacyclin therapy (intravenous or subcutaneous), escalation from subcutaneous to intravenous prostacyclin, or switching from phosphodiesterase‐5 inhibitors to soluble guanylate cyclase stimulators. This aligns with contemporary trial endpoint constructs where escalation and hospitalization represent major morbidity and treatment failure signals in PH. All patients underwent systematic follow‐up at 3‐to‐6‐month intervals through outpatient evaluations and telephone interviews.

### 2.5 Statistical Analysis

Continuous variables were presented as mean ± standard deviation (SD) for normally distributed data or median (interquartile range [IQR]) for skewed distributions, with group comparisons performed using independent samples *t* test or Mann–Whitney *U* test as appropriate. Categorical variables were expressed as frequencies (%) and analyzed with *χ* ^2^ tests. Spearman's correlation analysis was performed to assess the correlation between HFpEF probability and risk factors and comorbidities of LHD. Time‐to‐event outcomes were evaluated using Kaplan–Meier curves and the log‐rank test, and multivariable Cox proportional hazards regression assessed the independent prognostic value of HFpEF‐ABA categories. The results were expressed as hazard ratio (HR) with corresponding 95% confidence interval (95% CI). Restricted cubic splines were incorporated into the Cox models to assess the dose‐response relationship and potential non‐linearity. We further evaluated whether integrating HFpEF‐ABA improved the discrimination of the COMPERA 2.0 risk stratification tool for clinical worsening. Model discrimination was quantified through the Harrell's concordance index (C‐index), and reclassification improvement was assessed through net reclassification improvement (NRI) and integrated discrimination improvement (IDI) using the R software package “survIDINR.” Subgroup analyses tested interactions with sex, cardiovascular comorbidities (CAD, hypertension, OSA), and lifestyle factors (smoking, alcohol use). All analyses were conducted in IBM SPSS 26.0 (Armonk, NY), GraphPad Prism 10.0 (GraphPad Software, USA), and R 4.4.1 (R Foundation) with two‐tailed *α* = 0.05 defining significance.

## 3 Results

### 3.1 Baseline Characteristics

As shown in Figure 1, of the initial 512 patients with CTEPH, 46 were excluded: age <18 years (*n* = 3), missing baseline clinical data (*n* = 18), LVEF < 50% (*n* = 3), and loss to follow‐up (*n* = 22). The final sample comprised 466 patients, with median age of 57 years (IQR 49–65 years), and 49.4% were female. Among them, 52.4% (*n* = 244) and 9.0% (*n* = 42) of patients with CTEPH had intermediate and high HFpEF probabilities, respectively. As shown in Table 1, participants with clinical worsening (*n* = 115) were older, had a higher prevalence of AF, higher total and direct bilirubin and creatinine levels, and higher HFpEF‐ABA score compared with participants without clinical worsening (*p* < 0.05). In contrast, participants without clinical worsening had higher rates of pulmonary endarterectomy or balloon pulmonary angioplasty compared with those with clinical worsening (*p* < 0.05).

**Figure 1:**
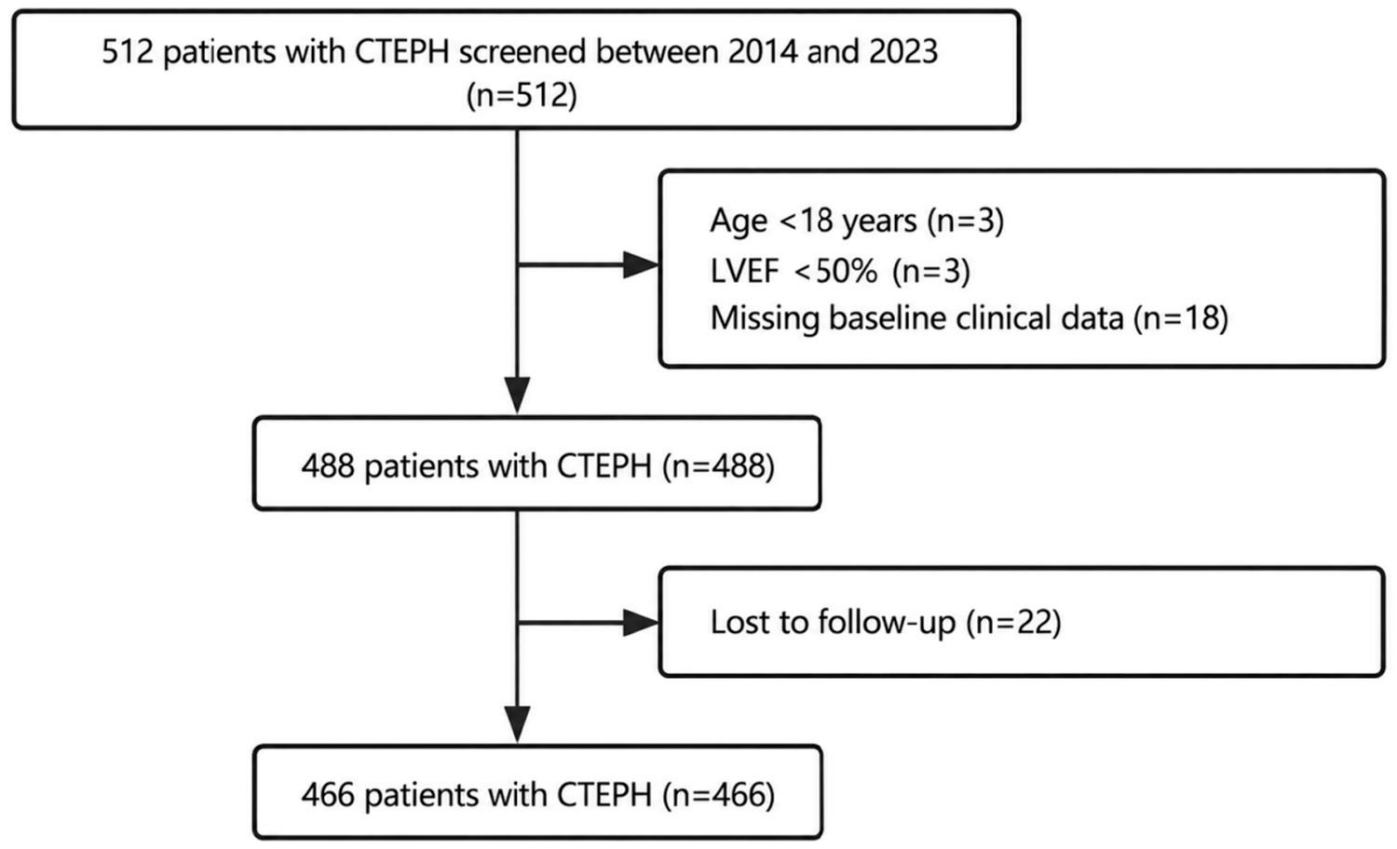
Flow diagram of patient's selection. CTEPH, chronic thromboembolic pulmonary hypertension; LVEF, left ventricular ejection fraction.

**Table 1:** Baseline characteristics of patients with and without clinical worsening.

Variables	All (*n* = 466)	Non‐clinical worsening (*n* = 351)	Clinical worsening (*n* = 115)	*p* value
Clinical characteristics				
Age, years	57 (49, 65)	57 (49, 64)	61 (53, 69)	< 0.001
Female, *n* (%)	230 (49.4%)	180 (51.3%)	50 (43.5%)	0.146
BMI, kg/m^2^	23.87 ± 3.57	23.50 ± 3.30	24.60 ± 4.00	0.053
Current smoking, *n* (%)	133 (28.5%)	94 (26.8%)	39 (33.9%)	0.142
Current drinking, *n* (%)	100 (21.5%)	73 (20.8%)	27 (23.5%)	0.543
Hypertension, *n* (%)	124 (26.6%)	87 (24.8%)	37 (32.2%)	0.120
DM, *n* (%)	112 (24.0%)	32 (27.8%)	80 (22.8%)	0.273
CAD, *n* (%)	37 (7.9%)	25 (7.1%)	12 (10.4%)	0.254
OSA, *n* (%)	116 (24.9%)	83 (23.6%)	33 (28.7%)	0.277
AF, *n* (%)	71 (15.2%)	39 (11.1%)	32 (27.8%)	< 0.001
Pericardial effusion, *n* (%)	81 (17.4%)	60 (17.1%)	21 (18.3%)	0.774
WHO FC				0.155
I–II, *n* (%)	209 (44.8%)	164 (46.7%)	45 (39.1%)	
III–IV, *n* (%)	257 (55.2%)	187 (53.3%)	70 (60.9%)	
Laboratory parameter				
NT‐proBNP, pg/mL	1069 (245, 2198)	971.9 (205.6, 2046.8)	1332.5 (342.8, 2543)	0.082
CRP, mg/L	3.15 (1.98, 5.53)	2.93 (1.92, 4.92)	3.82 (2.29, 6.66)	0.051
Albumn, g/L	42.06 ± 4.81	42.2 ± 4.78	42.13 ± 4.98	0.825
Creatine, μmol/L	83.83 (71.74, 96.00)	83.45 (71.00, 93.94)	87.95 (76.45, 102.05)	0.021
BUN, μmol/L	6.30 (5.25, 7.65)	6.25 (5.24, 7.57)	6.67 (5.25, 8.09)	0.186
Tbil, μmol/L	17.65 (12.92, 26.27)	16.85 (12.39, 24.40)	19.17 (13.53, 30.59)	0.030
Dbil, μmol/L	4.56 (3.01, 7.88)	4.33 (2.96, 7.44)	5.77 (3.20, 8.74)	0.043
ALT, U/L	22.00 (15.00, 32.00)	22.00 (15.75, 32.00)	22.00 (14.00, 34.00)	0.756
AST, U/L	27.00 (21.00, 33.00)	27.00 (22.00, 34.00)	26.00 (20.00, 33.00)	0.297
FBG, mmol/L	5.37 ± 1.34	5.38 ± 1.40	5.44 ± 1.07	0.497
HbA1_C_, %	6.13 ± 0.73	6.12 ± 0.77	6.18 ± 0.61	0.857
TC, mmol/L	4.3 (3.7, 5.0)	4.3 (3.7, 5.0)	4.3 (3.4, 5.2)	0.856
TG, mmol/L	1.2 (1.0, 1.6)	1.2 (0.9, 1.5)	1.3 (1.0, 2.0)	0.079
HDL‐C, mmol/L	1.2 (1.0, 1.5)	1.2 (1.0, 1.5)	1.1 (1.0, 1.5)	0.292
LDL‐C, mmol/L	2.6 (2.0, 3.2)	2.6 (2.0, 3.2)	2.6 (1.9, 3.5)	0.654
Echocardiography				
LA, mm	35.3 ± 5.9	35.3 ± 6.0	35.6 ± 6.1	0.831
LVEDD, mm	42.1 ± 6.9	42.3 ± 6.9	41.0 ± 6.8	0.422
RVEDD, mm	33.0 ± 7.0	33.3 ± 6.9	33.6 ± 7.4	0.842
LVEF, %	65.1 ± 5.6	65.7 ± 6.1	63.7 ± 4.6	0.057
TAPSE, mm	16 (14, 19)	16 (14, 19)	16 (13, 19)	0.572
TRV, m/s	4.1 ± 0.9	4.1 ± 1.0	4.2 ± 0.8	0.502
sPAP, mmHg	87.00 (69.00, 102.00)	86.00 (68.00, 102.50)	80.00 (74.00, 95.00)	0.491
RHC				
S_V_O_2_, %	68.0 ± 6.4	68.1 ± 6.6	67.8 ± 5.8	0.851
mRAP, mmHg	6 (4, 9)	6 (4, 9)	7 (4, 11)	0.264
mPAP, mmHg	48 (40, 57)	48 (41, 56)	45 (39, 55)	0.738
PAWP, mmHg	9 (7, 12)	9 (6, 12)	10 (8, 13)	0.052
RVR, WU	9.15 (5.91, 12.69)	10.16 (6.78, 13.20)	8.36 (5.91, 12.82)	0.203
Cardiac index, L/min	2.83 (2.31, 3.35)	2.86 (2.39, 3.36)	2.69 (2.12, 3.24)	0.387
Treatment				
PEA or BPA, *n* (%)	231 (49.7%)	184 (52.6%)	47 (40.9%)	0.029
PH‐targeted medicine, *n* (%)	338 (72.5%)	253 (72.1%)	85 (73.9%)	0.702
HFpEF‐ABA score (per SD)	29.78 (18.66, 43.04)	28.29 (17.83, 40.40)	37.44 (21.13, 58.62)	< 0.001
HFpEF‐ABA score (tertile)				< 0.001
Low probability	180 (38.6%)	145 (41.3%)	35 (30.5%)	
Intermediate probability	244 (52.4%)	189 (53.8%)	55 (47.8%)	
High probability	42 (9.0%)	17 (4.9%)	25 (21.7%)	

Abbreviations: AF, atrial fibrillation; ALT, alanine aminotransferase; AST, aspartate transaminase; BMI, bodymass index; BUN, blood urea nitrogen; BPA, balloon pulmonary angioplasty; CI, confidence interval; CRP, C‐reactive protein; CAD, coronary artery disease; DM, diabetes mellitus; Dbil, direct bilirubin; FBG, fasting blood glucose; HbA1c, glycated hemoglobin; HDL‐C, high density lipoprotein cholesterol; HFpEF, Heart failure with preserved ejection fraction; HFpEF‐ABA score, a clinical model using age, body mass index and history of atrial fibrillation; LA, left atrial; LVEF, left ventricular ejection fraction; LVEDD, left ventricular end‐diastolic internal diameter; LDL‐C, low density lipoprotein cholesterol; mRAP, mean right atrial pressure; mPAP, mean pulmonary arterial pressure; NT‐proBNP, N‐terminal pro‐B‐type natriuretic peptide; OSA, obstructive sleep apnea; PH, pulmonary hypertension; PEA, pulmonary endarterectomy; PVR, pulmonary vascular resistance; PAWP, pulmonary arterial wedge pressure; RVEDD, right ventricular end‐diastolic internal diameter; RHC, right heart catheterization; SD, standard deviation; S_V_O_2_, saturation of mixed venous blood oxygen; sPAP, pulmonary artery systolic pressure; ‌Tbil, total bilirubin; TC, total cholesterol; TG, triglyceride; TAPSE, tricuspid annular plane systolic excursion; TRV, tricuspid regurgitation velocity; WHO FC, World Health Organization Functional Class.

### 3.2 Relationship Between HFpEF‐ABA Score and Common LHD Comorbidities

As detailed in Table 2, the HFpEF‐ABA score demonstrated significant associations with LHD‐related comorbidity burden. Significant positive correlations were observed between the HFpEF‐ABA score and LHD risk factors: obesity (*r* = 0.202), hypertension (*r* = 0.235), CAD (*r* = 0.156), and OSA (*r* = 0.244) (*p* < 0.05).

**Table 2:** Correlation analysis of HFpEF‐ABA score with common left heart disease comorbidities.

Common LHD comorbidities		
Variables	Coefficient (*r*)	*p* value
Obesity	0.202	< 0.001
Hypertension	0.235	< 0.001
Coronary artery disease	0.156	< 0.001
OSA	0.244	< 0.001

Abbreviations: CTEPH, chronic thromboembolic pulmonary hypertension; HFpEF, heart failure with preserved ejection fraction; HFpEF‐ABA score, a clinical model using age, body mass index and history of atrial fibrillation; LHD, left heart disease; OSA, obstructive sleep apnea.

### 3.3 Predictive Value of HFpEF‐ABA Score for Clinical Worsening

Over a median follow‐up of 27 months (IQR 13–45 months), clinical worsening occurred in 115 patients (24.7%). Kaplan–Meier analysis showed that patients with a high probability of HFpEF (≥ 75%) had a significantly lower event‐free survival than those with intermediate (25%–74%) and low (< 25%) probability (log‐rank *p* < 0.0001; Figure 2A). This association remained consistent when patients were dichotomized according to the high HFpEF probability threshold of ≥ 75% (Figure 2B).

**Figure 2:**
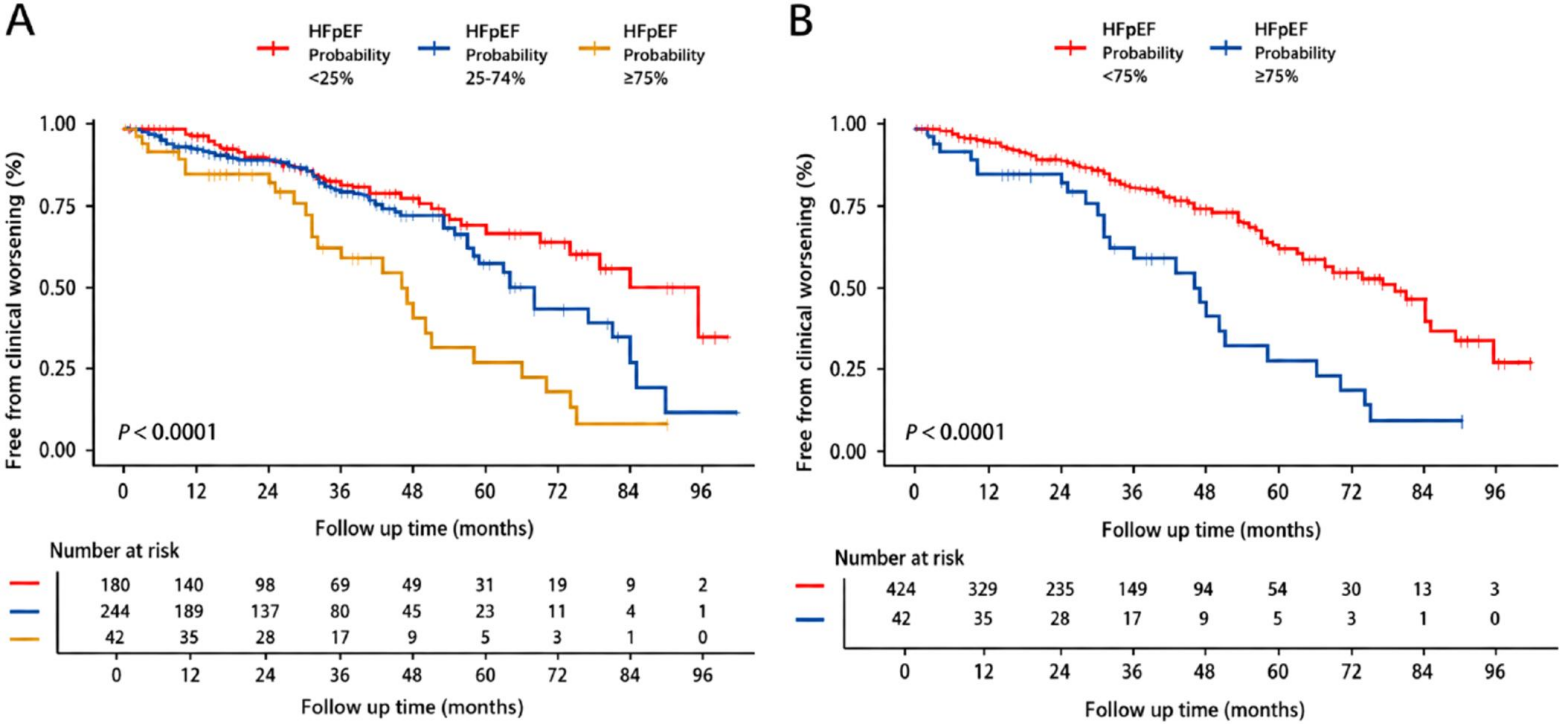
Kaplan–Meier estimated event rates of clinical worsening according to HFpEF‐ABA score. (A) CTEPH patients with a high probability of HFpEF (≥ 75%) had significantly lower event‐free survival than CTEPH patients with a moderate (25%–74%) or low (< 25%) probability of HFpEF, respectively. (B) CTEPH patients with a high probability of HFpEF (≥ 75%) had significantly lower event‐free survival than CTEPH patients with a medium‐low probability of HFpEF (< 75%). HFpEF, heart failure with preserved ejection fraction; HFpEF‐ABA score, a clinical model using age, body mass index and history of atrial fibrillation.

To validate the independent prognostic value of the HFpEF‐ABA score, we performed multivariable Cox regression analyses with progressive adjustment for potential confounders, as shown in Table 3. Variables included in the multivariable models were selected based on clinical relevance, their established prognostic importance in CTEPH risk stratification, and model stability. Model 1 was adjusted for sex; because age is included in the HFpEF‐ABA algorithm, age was not additionally entered as a separate covariate in this model to avoid potential collinearity and overadjustment; Model 2 was further adjusted for ln (NT‐proBNP), World Health Organization functional class, 6‐min walk distance, balloon pulmonary angioplasty or pulmonary endarterectomy, and PH‐specific treatment; and Model 3 was additionally adjusted for mean right atrial pressure, saturation of mixed venous blood oxygen, cardiac index, and PVR. The base model (Model 1), adjusted for sex, demonstrated that each SD increase in HFpEF‐ABA score was associated with a 53.5% elevated risk of clinical worsening (HR 1.535, 95% CI 1.319–1.787, *p* < 0.001). In the fully adjusted model (Model 3), each SD increase in the HFpEF‐ABA score was associated with a 73.0% higher risk of clinical worsening (HR 1.730, 95% CI 1.368–2.188, *p* < 0.001).

**Table 3:** Multivariate Cox analysis of HFpEF pretest probability and clinical worsening in CTEPH patients.

	Model 1		Model 2		Model 3	
Variables	HR (95% CI)	*p* value	HR (95% CI)	*p* value	HR (95% CI)	*p* value
Continuous						
HFpEF‐ABA score (per SD)	1.535 (1.319–1.787)	< 0.001	1.547 (1.271–1.883)	< 0.001	1.730 (1.368–2.188)	< 0.001
Dichotomy						
HFpEF‐ABA score (< 75%)	—		—		—	
HFpEF‐ABA score (≥ 75%)	2.686 (1.715–4.206)	< 0.001	2.392 (1.426–4.010)	< 0.001	2.836 (1.571–5.119)	< 0.001
Three‐category						
HFpEF‐ABA score (< 25%)	—		—		—	
HFpEF‐ABA score (25%–74%)	1.519 (0.989–2.334)	0.056	1.243 (0.743–2.078)	0.408	1.306 (0.712–2.395)	0.388
HFpEF‐ABA score (≥ 75%)	3.409 (2.024–5.740)	< 0.001	2.782 (1.476–5.245)	0.002	3.464 (1.634–7.344)	0.001

*Note:* Model 1: Adjusted for sex; Model 2: Model 1 plus ln (NT‐proBNP); WHO FC; 6MWD, BPA or PEA; PH‐specific treatment; Model 3: Model 2 plus mRAP, S_V_O_2;_ cardiac index; PVR.

Abbreviations: BPA, balloon pulmonary angioplasty; CTEPH, chronic thromboembolic pulmonary hypertension; CI, confidence interval; HFpEF, heart failure with preserved ejection fraction; HFpEF‐ABA score, a clinical model using age, body mass index and history of atrial fibrillation; HR, hazard ratio; mRAP, mean right atrial pressure; 6MWD, 6‐min walk distance; NT‐proBNP, N‐terminal pro brain natriuretic peptide; PVR, pulmonary vascular resistance; PEA, pulmonary endarterectomy; PH, pulmonary hypertension; SD, standard deviation; S_V_O_2_, saturation of mixed venous blood oxygen; WHO‐FC, World Health Organization functional class.

Consistent results were observed when the HFpEF‐ABA score was modeled as a dichotomous variable. Compared with patients with HFpEF‐ABA score < 75%, those with HFpEF‐ABA score ≥ 75% had a significantly higher risk of clinical worsening in Model 1 (HR 2.686, 95% CI 1.715–4.206, *p* < 0.001), Model 2 (HR 2.392, 95% CI 1.426–4.010, *p* < 0.001), and Model 3 (HR 2.836, 95% CI 1.571–5.119, *p* < 0.001). When the HFpEF‐ABA score was modeled as a three‐category variable, the high‐probability group (≥ 75%) remained significantly associated with clinical worsening compared with the low‐probability group (< 25%) across all three models, including Model 3 (HR 3.464, 95% CI 1.634–7.344, *p* = 0.001). In contrast, the intermediate‐probability group (25%–74%) was not significantly associated with clinical worsening compared with the low‐probability group. Overall, the HFpEF‐ABA score maintained its predictive capacity regardless of whether it was modeled as a continuous, dichotomous, or three‐category variable (Table 3). Restricted cubic spline analysis revealed a linear dose‐response relationship between HFpEF‐ABA score and clinical worsening risk (*p* for non‐linearity = 0.732, Figure 3), with progressively higher event rates observed at elevated score levels.

**Figure 3:**
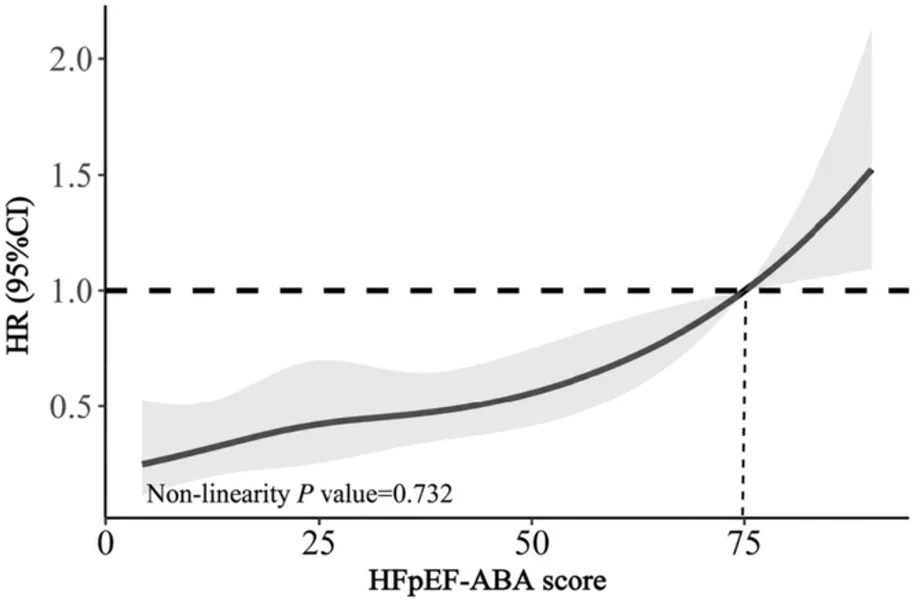
Restricted cubic spline curves for clinical worsening by HFpEF‐ABA score. HFpEF‐ABA scores showed a linear relationship with clinical deterioration, and CTEPH patients with higher scores had a higher risk of clinical deterioration. CI, confidence interval; HFpEF, heart failure with preserved ejection fraction; HFpEF‐ABA score, a clinical model using age, body mass index and history of atrial fibrillation; HR, hazard ratio.

### 3.4 HFpEF‐ABA Score Incremental Predictive Value

Adding the HFpEF‐ABA score improved the C‐index for COMPERA 2.0 risk score (0.532 vs. 0.634, *p* = 0.012). Incorporating the HFpEF‐ABA score into the COMPERA 2.0 score produced statistically significant improvements in reclassification accuracy, as demonstrated by significant NRI 0.345 (*p* < 0.001) and IDI 0.081 (*p* < 0.001).

### 3.5 Subgroup Analysis

Subgroup analyses were performed, stratifying patients with CTEPH by sex, smoking and alcohol use, hypertension, CAD, and OSA, as shown in Figure 4. No significant interactions were observed across subgroups (*p* for interaction > 0.05), and the effect of the HFpEF‐ABA score in predicting the risk of clinical worsening was consistent across subgroups.

**Figure 4:**
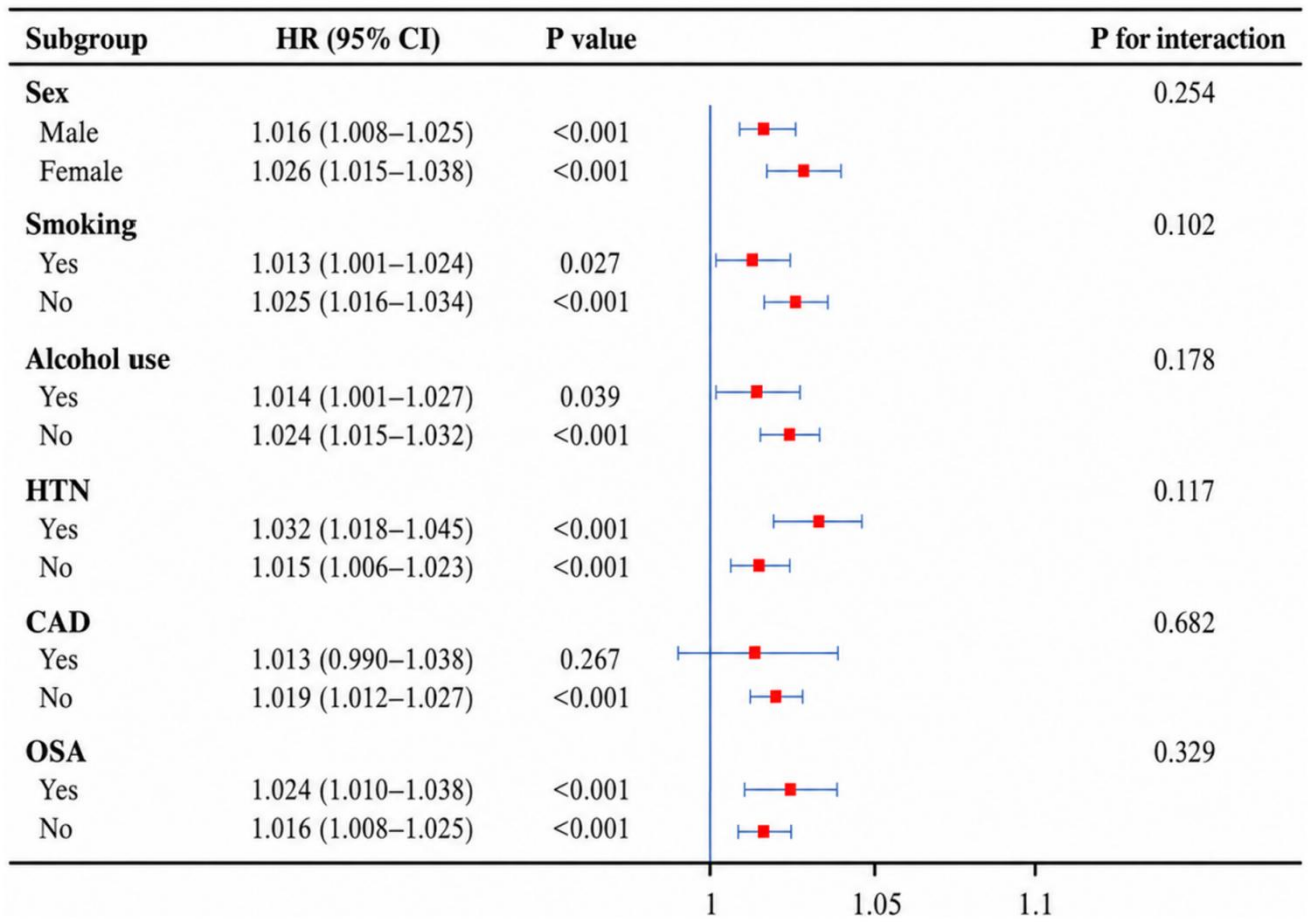
Association between HFpEF‐ABA score (per SD increase) and clinical worsening in different subgroups. There was no significant interaction in any subgroup (*p* > 0.05 for interaction); the effect of the HFpEF‐ABA score in predicting the risk of clinical deterioration was identical in each subgroup. CAD, coronary artery disease; CI, confidence interval; HFpEF, heart failure with preserved ejection fraction; HFpEF‐ABA score, a clinical model using age, body mass index and history of atrial fibrillation; HR, hazard ratio; OSA, obstructive sleep apnea; SD, standard deviation.

## 4 Discussion

In the current study we found that (1) 52.4% patients with CTEPH had a moderate probability of HFpEF and 9.0% had a high probability of HFpEF; (2) elevated HFpEF‐ABA score was positively associated with LHD comorbidities and risk factors (obesity, hypertension, CAD, OSA) (*p* < 0.05); (3) elevated HFpEF‐ABA score independently predicted clinical worsening events in patients with CTEPH, linking the HFpEF‐ABA score in combination with the COMPERA 2.0 risk score significantly improved their prediction, reclassification and identification.

### 4.1 Impact of LHD and Its Risk Factors on Patients With CTEPH

Age is a major risk factor for cardiovascular and metabolic diseases [17]. Previous studies have reported a median age of approximately 59–63 years in patients with CTEPH, and the median age of our cohort was 57 years, which is broadly consistent with previous CTEPH cohorts. Importantly, CTEPH commonly occurs within an age range in which LHD‐ and HFpEF‐related comorbidities become increasingly prevalent [18, 19]. Therefore, although CTEPH and HFpEF represent distinct clinical entities, the potential coexistence of LHD‐ or HFpEF‐related features should be considered during clinical assessment.

In fact, it has been shown that 10.6%–37.4% of patients with CTEPH also present with manifestations of elevated left ventricular filling pressure [20]. Potential cardiac comorbidities may further complicate the identification of PH etiology. One study included 55 patients with CTEPH treated with riociguat, of whom 40 subjects (91%) had at least one definite cardiopulmonary comorbidity and 19 subjects (40%) had two or more HFpEF risk factors [21]. GERGES et al. [20] demonstrated that among CTEPH patients, 11% exhibited elevated left ventricular filling pressures when applying a threshold of > 15 mmHg, while 37% showed elevated pressures at a threshold of > 11 mmHg. These patients were characterized by older age, a higher prevalence of hypertension, DM, AF, and larger left atrial volumes compared to those without elevated filling pressures.

In our study, we used the HFpEF‐ABA score, which has been validated by Reddy et al. [10] for predicting the probability of HFpEF. Among patients with CTEPH, 52.4% had an estimated HFpEF probability of 25%–74%, and 9.0% had a probability of ≥ 75%.

### 4.2 HFpEF: A Common LHD Outcome

The definition of HFpEF requires heart failure symptoms/signs, a LVEF ≥ 50%, and either structural/functional cardiac abnormalities or elevated natriuretic peptides. However, diagnosing HFpEF is challenging because about 20% of patients have normal natriuretic peptide levels, making it impossible to rely solely on imaging or a simple peptide cutoff [7]. HFpEF is likely to be the most common type of heart failure, in part because it is a problem specific to the elderly and because of the global ageing of the population, and advancing age accrues comorbidities [22]. Multiple comorbidities, including hypertension, CAD, obesity, OSA, DM, chronic kidney disease, and AF, contribute to HFpEF pathogenesis and progression [23, 24]. The cumulative burden of these conditions correlates with more severe symptomatic presentation, reduced quality of life, and worse clinical outcomes. Obesity plays a central role in HFpEF pathophysiology, with epidemiological studies demonstrating elevated risk among obese individuals and symptom improvement following weight reduction strategies [25, 26]. AF and HFpEF frequently coexist, with bidirectional pathophysiological interactions—AF represents the most prevalent arrhythmia in heart failure populations, while heart failure accounts for the leading cause of mortality in AF patients. Notably, subclinical HFpEF was detected in 70% of AF patients post‐catheter ablation despite absent overt heart failure symptoms, as evidenced by Australian cohort data [27].

### 4.3 Predictive Value of Pretest Probability of HFpEF for Prognosis in Patients With CTEPH

HFpEF is frequently underdiagnosed in clinical practice, while current diagnostic algorithms typically require echocardiographic and invasive catheterization confirmation [28]. Based on the above research evidence, Reddy et al. [10, 20] developed the HFpEF‐ABA score utilizing three easily obtainable clinical parameters: age, BMI, and history of AF. This score demonstrated robust predictive capability (derivation cohort area under the curve 0.84, validation cohorts' area under the curve 0.81–0.93) and effectively identified high‐risk patients in real‐world populations. Subsequent application in PAH patients revealed that the HFpEF‐ABA score could detect individuals with subclinical LHD, characterized by poor dynamic PAWP response, impaired functional status, reduced quality of life, and worse survival outcomes [11]. CTEPH, classified as Group 4 PH, shares hemodynamic similarities with PAH (Group 1), as both conditions manifest as precapillary PH [29]. Our study further validated the predictive value of elevated HFpEF‐ABA score for clinical worsening events in patients with CTEPH, obtaining similar results to the study by Reddy et al. [11], and challenges the conventional dichotomous classification separating HFpEF from CTEPH, instead proposing a continuous risk spectrum quantifiable through three routinely available clinical variables. Notably, in our study, incorporating the HFpEF‐ABA score improved both the performance of the COMPERA 2.0 model, potentially refining diagnostic accuracy and therapeutic decision‐making for patients with overlapping cardiovascular comorbidities. Our continuum‐based approach offers superior risk stratification compared to existing categorical models, suggesting that HFpEF‐ABA assessments can enhance current risk prediction frameworks for CTEPH. These findings represent significant progress beyond oversimplified classification schemes currently employed in clinical practice.

### 4.4 Interaction Between CTEPH and HFpEF in Pathophysiological Mechanisms

Several potential mechanisms may explain the association between higher HFpEF probability and adverse outcomes in patients with CTEPH. Patients with pulmonary hypertension due to LHD, particularly HFpEF, often have elevated left ventricular filling pressure, which can be transmitted backward to the pulmonary veins and lead to postcapillary pulmonary hypertension. Over time, persistently increased pulmonary venous pressure may promote pulmonary vascular remodeling, increase PVR, and contribute to combined precapillary and postcapillary pulmonary hypertension. In patients with CTEPH, the primary hemodynamic abnormality is precapillary pulmonary vascular obstruction caused by organized thromboembolic lesions and secondary microvasculopathy. However, CTEPH patients may also have clinical risk factors associated with PH‐LHD, including older age, obesity, metabolic syndrome, hypertension, atrial fibrillation, and ischemic heart disease [18, 30]. These factors are closely related to left ventricular systolic and/or diastolic dysfunction and elevated left ventricular filling pressure. Therefore, in patients with a high HFpEF probability, a postcapillary component may coexist with the precapillary thromboembolic burden, resulting in a more complex and severe hemodynamic phenotype.

In addition, right ventricular pressure overload in CTEPH may itself adversely affect left ventricular filling. Increased right ventricular pressure and volume load can induce interventricular septal shift, impair left ventricular diastolic function, reduce left ventricular preload, and cause left ventricular underfilling. These changes may be particularly important in patients with underlying HFpEF susceptibility, in whom left ventricular relaxation and compliance are already impaired. Thus, CTEPH and HFpEF may interact through both pulmonary vascular and ventricular interdependence mechanisms.

Patients with overlapping CTEPH‐HFpEF features may therefore present with a “double‐hit” pathophysiological phenotype, characterized by proximal thromboembolic obstruction, distal pulmonary microvasculopathy, and elevated left ventricular filling pressure. This combined burden may aggravate pulmonary vascular remodeling, impair right ventricular adaptation, reduce cardiopulmonary reserve, and contribute to worse clinical outcomes. From this perspective, CTEPH and HFpEF should not be viewed as completely separate entities in clinical practice. Instead, a holistic assessment of both precapillary thromboembolic disease and HFpEF‐related cardiometabolic comorbidities may be important for risk stratification.

Our study demonstrated the prognostic value of HFpEF probability in patients with CTEPH and showed that adding HFpEF‐ABA to traditional CTEPH risk stratification improved predictive performance. The above pathophysiological mechanisms may help explain these findings. Moreover, patients with overlapping CTEPH‐HFpEF features may have residual symptoms or incomplete improvement after CTEPH‐directed therapy, because elevated left ventricular filling pressure and HFpEF‐related comorbidities may persist even after treatment of the precapillary thromboembolic component. Therefore, comprehensive and individualized management, including CTEPH‐specific evaluation and optimization of HFpEF‐related comorbidities, may be needed in this phenotype [31, 32]. Riociguat is an established therapy for selected patients with CTEPH. The DYNAMIC trial showed that riociguat improved selected hemodynamic parameters in patients with PH‐HFpEF. These findings suggest that riociguat may have potential therapeutic relevance for CTEPH patients with concomitant HFpEF‐related features, although its clinical benefit in this overlapping phenotype requires further investigation [33]. Further prospective studies are warranted to clarify whether specific treatment strategies can improve outcomes in patients with overlapping CTEPH‐HFpEF features.

### 4.5 Study Limitations

There are some limitations. First, as a single‐center retrospective study, our findings are potentially limited by unmeasured confounding and selection bias. Although multivariable adjustment was performed, residual confounding from unmeasured variables, such as medication adherence, socioeconomic status, health‐care access, and longitudinal changes in treatment strategies, cannot be fully excluded. Therefore, our findings require external validation and warrant confirmation in larger prospective cohorts. Second, our findings should be interpreted as indicating a high pre‐test probability of HFpEF rather than confirmed left heart dysfunction, and future studies incorporating direct measures of left ventricular filling pressures are needed to validate this association. Finally, whether patients with high HFpEF probability require treatment modification needs to be further investigated.

## 5 Conclusion

This study establishes the HFpEF‐ABA score as a clinically meaningful predictor of LHD burden and prognosis in CTEPH. The score's independent prognostic value and ability to improve COMPERA 2.0 model performance highlight the importance of assessing concomitant cardiac comorbidities in CTEPH risk stratification. These findings advocate for routine HFpEF probability evaluation in CTEPH management and warrant investigation of phenotype‐specific therapies.

## Author Contributions

Zhihong Liu, Qin Luo, and Zhihui Zhao provided contributions to the conceptualization and design of the study. Yijia Wang, Xin Li, Qi Wang, and Tao Yang were responsible for data collection. Statistical analysis was conducted by Yijia Wang, Xin Li, Zhihua Huang, Sicong Li, and Luyang Gao. Yijia Wang and Xin Li drafted the manuscript, while Qing Zhao, Qin Luo, Zhihui Zhao, and Zhihong Liu reviewed and revised it. Yijia Wang, Xin Li, Anqi Duan, and Qi Wang accessed and verified the data. All authors reviewed and ratified the final manuscript.

## Ethics Statement

The research adhered to the Declaration of Helsinki and obtained approval from the Fuwai Hospital Ethics Committee (No. 2024‐2441).

## Conflicts of Interest

The authors declare no conflicts of interest.

## Data Availability

The data analyzed in this study are available on reasonable request from the corresponding authors.
